# Smartphone-Assisted Glaucoma Screening in Patients With Type 2 Diabetes: a Pilot Study

**Published:** 2020-01-01

**Authors:** Yannick Bilong, Christelle Noche Domngang, Gebding Nwanlih Gimma, Jean-Claude Katte, Ted Evina Afetane, Gilles Kagmeni, Jean Claude Mbanya, Nilesh Kumar, Ashish Sharma, Eugene Sobngwi

**Affiliations:** 1 Department of Ophthalmology, Faculty of Medicine and Biomedical Sciences, The University of Yaounde 1, Yaounde, Cameroon; 2 Department of Clinical Sciences, Faculty of Health Sciences, Universite des Montagnes, Bangangte, Cameroon; 3 Department of Public Health, Faculty of Medicine and Biomedical Sciences, The University of Yaounde 1, Yaounde, Cameroon; 4 Ophthalmo-Pediatric Unit, Magrabi ICO Cameroon Eye Institute, Okala, Cameroon; 5 Department of Internal Medicine and Specialties, Faculty of Medicine and Biomedical Sciences, University of Yaounde 1, Yaounde, Cameroon; 6 Department of Vitreoretina, Lotus Eye Hospital and Institute, Coimbatore, TN, India

**Keywords:** Smartphone, Glaucoma, Screening, Diabetes, Make in India Retinal Camera (MIIRetCam)

## Abstract

We aimed to determine true and false positives of glaucoma screening, relying solely on photos of the retina, taken with a smartphone. We performed a descriptive and analytical study on patients with type 2 diabetes at the National Obesity Centre, Yaoundé, Cameroon. Participating patients had retinal photography sessions using an iPhone 5s (iOS 10.3.3; Apple, Cupertino, CA) coupled to the Make in India Retinal Camera (MIIRetCam; MIIRetCam Inc., Coimbatore, TN, India). Obtained pictures of the retina were stored and transferred via the internet to an ophthalmologist to assess glaucoma. Selected patients were then invited to undergo a conventional ophthalmological examination to confirm the diagnosis. A total of 395 patients were screened, 39 (including 20 women) were diagnosed with suspicion of glaucoma based on retinal photos, a prevalence rate of 9.87%. The following signs were found; Cup/Disc ratio (C/D) ≥0.5 in 64.1% (25/39), asymmetric C/D >0.2 in 35.9% (14/39), papillary haemorrhage in 10.2% (4/39) and retinal nerve fibre deficiency in 2.5% (1/39). Only 14 of 39 patients with suspicion of glaucoma were examined, giving a lost-to-follow-up rate of 64.1%. Chronic open-angle glaucoma was confirmed in 8 patients (true positives) and absent in 6 patients (false positives). The prevalence of smartphone-detected glaucoma and lost-to-follow-up rates were high. So we need to improve this type of screening, with additional tests like transpalpebral applanation tonometer and the smartphone Frequency Doubling Technique visual field combined with better education of patients to increase their adherence to follow-up.

## INTRODUCTION

Glaucoma is one of the leading causes of blindness worldwide but its diagnosis remains a public health challenge, especially in developing countries [1]. Community screening for glaucoma is becoming an important topic in ophthalmology given the long asymptomatic nature of the disease, which underpins the progressive loss of retinal ganglion fibres[2, 3]. Despite the fact that screening of glaucoma has not been standardised, coherent principles are outlined and adapted according to technical platform and socioeconomic context within two successive stages [4, 5]. 

The first stage involves risk stratification of the population and qualitative analysis of the optic nerve. Several risk factors for glaucoma exist including advanced age, male, genetics and family history, myopia, hypertension and diabetes [6]. The qualitative analysis of the optic nerve involves the use of available technologies (direct or indirect ophthalmology, traditional or portable fundus camera), and possibly the use of a reliable, rapid, reproducible functional and quantifiable study of the optic nerve using the Frequency Doubling Technique (FDT) [7]. 

In the second stage of screening, patients with a suspicion of glaucoma will then undergo a conventional ophthalmological examination to confirm or rule out the diagnosis of glaucoma. To develop a screening method accessible to all, and at a lower cost, we performed a pilot study using smartphone-assisted retinal photography to visualise and analyse the optic disc. This process of examining the optic nerve papilla had been previously demonstrated by Russo et al. as being identical to that performed by slit lamp [8]. 

The objective of this study was to determine the rates of true and false positives in a group of patients with type 2 diabetes, who were imaged for diabetic retinopathy screening with an initial suspected diagnosis of glaucoma on retinal photos using smartphone. 

## METHODS

We performed a descriptive and analytical study during a period of 8 months (01 January to 30 August 2018). This study was performed at two locations involving a screening site and a confirmation site. The screening site was the diabetes clinic out-patient department of the National Obesity Centre at the Yaoundé Central Hospital, Cameroon. Patients with suspicion of glaucoma were then invited to the Ophthalmology Department of the Yaoundé Gynaeco-Obstetric and Paediatric Hospital for confirmation of diagnosis.

Adult patients with type 2 diabetes were invited to participate in the study and provided a written informed consent before retinal photography. Ethical approval for the study was obtained from the National Ethics Committee. Patients who had hazy optical media of the anterior segment in at least one eye, making a fundus evaluation impossible were excluded from the study. 

Once the patient was selected, retinal photos were taken using an iPhone 5s (iOS 10.3.3; Apple, Cupertino, CA) with a 20 diopters lens attached to the Make in India Retinal Camera (MIIRetCam; MIIRetCam Inc., Coimbatore, TN, India). The picture was taken after dilatation of the pupil with tropicamide 0.5% (Mydriaticum® 0.5%, eye drops, Thea Pharma laboratories, Inc.), one drop and if needed repeated after 30 minutes. The operator was a general practitioner trained in this imaging technique. At least one photo per eye of the papilla, was required. Uncompressed photos were saved on the cloud using in-built internet capabilities of the smartphone. An ophthalmologist later studied the pictures on the screen of the iPhone 5s (iOS 10.3.3; Apple, Cupertino, CA), and thereby diagnosed patients with suspicion of glaucoma, based on presence of at least one of the following criteria; Vertical Cup/disc (C/D) ratio ≥0.5; vertical C/D ratio asymmetry >0.2; presence of papillary haemorrhage or retinal nerve fibre deficiency.

All patients suspected of having glaucoma after their retinal photo analysis by the ophthalmologist were invited for the confirmation of diagnosis with full set of examination including tonometry (Icare TA01 tonometer), pachymetry (PachPen - Accutome), gonioscopy (Volk, 03 mirrors lens) and visual field (Octopus 300 V5.17) studies. The true positive and false-positive rates of smartphone imaging were determined. Results are presented as frequencies or mean and standard deviation. Data analysis was done using the EPI Info software version 3.5.4.

## RESULTS

We examined 395 patients with type 2 diabetes, with a mean ± standard deviation (SD) of age of 59 ± 7.6 years and a mean ± SD of duration of diabetes of 8 ± 5 years. Women made up 51.3% of patients (203/395 patients). 

**Figure 1:**
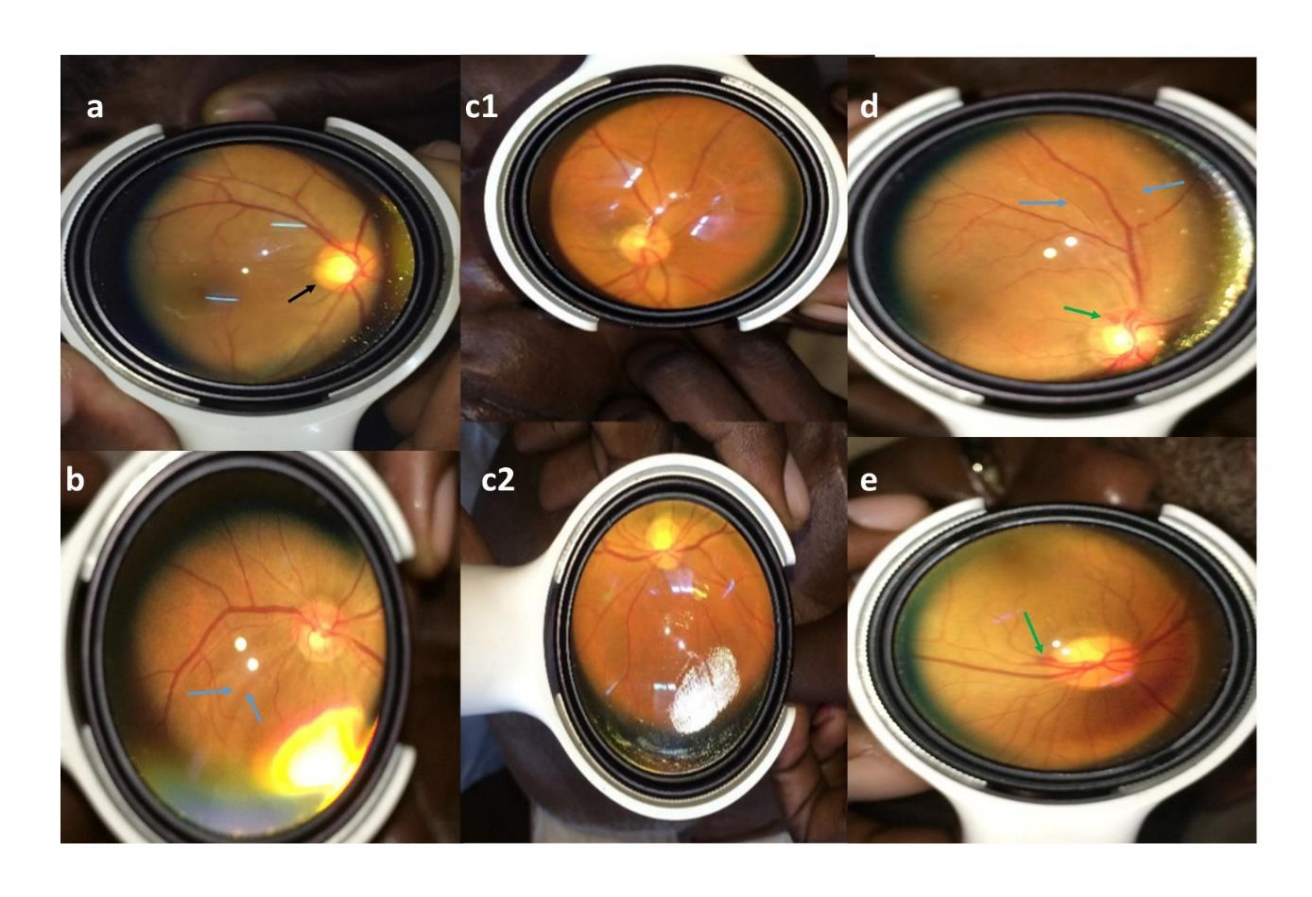
A = Cup/disc (C/D) vertical ratio ≥0.5 (black arrow); B= Retinal nerve fibre deficiency (blue arrow) ; C1 and C2 = Asymmetry of vertical C/D ratio >0.2; D= Presence of papillary haemorrhage (green arrow) associated to retinal nerve fibre deficiency (blue arrow); and E= presence of isolated papillary haemorrhage (green arrow).

The rate of suspicion of glaucoma on retinal photography was 9.87% (39/395 patients) based on qualitative changes in the optic nerve (Figure 1). The suspicion of glaucoma was mainly based on presence of a vertical C/D ratio ≥ 0.5. This sign was isolated in 15 patients and associated in 10 patients with vertical C/D ratio asymmetry >0.2. Other isolated signs were observed in 3 patients with vertical C/D ratio asymmetry >0.2 and 3 patients with papillary haemorrhage. Only one patient had combined 3 signs of vertical C/D ratio asymmetry >0.2, presence of papillary haemorrhage and retinal nerve fibre deficiency (Figure 1 B-D).

**Figure 2:**
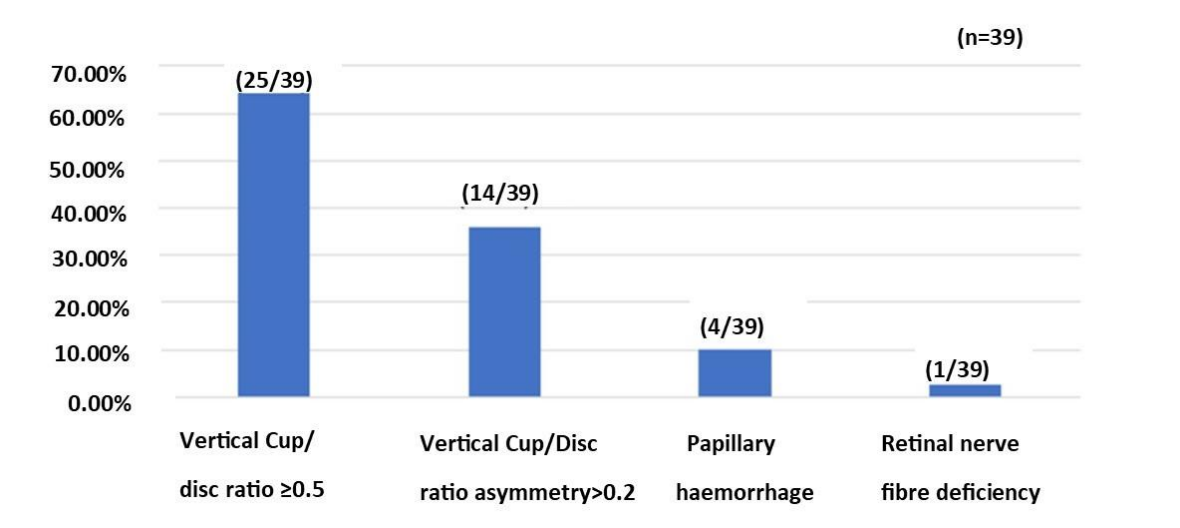
Clinical Signs to Suggest Glaucoma in Retinal Photos Taken Using an iPhone 5s in Study Subjects.

**Table 1:** Clinical Findings of Patients With Confirmed Glaucoma Following Smartphone-Assisted Glaucoma Screening

	Patients (n=8)
Age (Up to 50 years)	07
Female	06
Asymptomatic	08
Visual acuity (6/6)	08
Gonioscopy (Open angle)	08
Thin cornea (less than 500 micrometers)	05
Normal cornea (500-560 micrometers)	03
Vertical Cup/disc (C/D) ratio ≥0.5	01
Vertical C/D ratio ≥0.5 + asymmetry >0.2	03
Papillary haemorrhage	03
Vertical C/D ratio asymmetry >0.2 + papillary haemorrhage + retinal nerve fibre deficiency	01
Abnormal visual field	05

Fourteen patients (14/39) with initial suspicion of glaucoma underwent diagnostic ophthalmological examinations, so lost-to-follow-up rate was 64% (25/39). Diagnosis of glaucoma was confirmed in 8 of these 14 patients (Table 1). All of them (8/8) had chronic open angle glaucoma with a half (4/8) having high intraocular pressure (25 ± 3mmHg). The cornea was thin in five patients (481 ± 3 microns). Visual fields were abnormal in five patients, 03 had nasal defects and 02 had Bjerrum scotomas.

## DISCUSSION

In this pilot study, we demonstrated the possibility of screening for glaucoma using a smartphone-assisted retinal photographic technique. More than a half (8/14) of participants with initial suspicion of glaucoma who attended the confirmation examination were confirmed to have glaucoma. 

The use of smartphone for retinal pathology photography is a recent technique accessible to a wide range of healthcare practitioners and is inexpensive. It is already used in the screening of diabetic retinopathies [9] and retinopathy of prematurity [10]. In glaucoma screening, the smartphone is now a multi-tasking tool for examining the optic nerve [8], the iridocorneal angle [11] and the FDT visual field [12]. In the near future it could enable the examination of intraocular pressure[13].

Examination of papilla in isolation is not an early screening tool for glaucoma, due to its low sensitivity and should be combined with other tests [14]. This implies that our 9.87% screening rate would actually be higher if other tests were added. A population-wide Chinese study using the same criteria for optic nerve analysis had a glaucoma suspicion rate of 1.93% [5]. Our small sample population comprising of patients with diabetes, older and Africans sub-Saharans ones with a higher risk of glaucoma could justify such different rates. Nevertheless, our study had the strength of bringing to light advanced stages of glaucoma in a population at risk, which would otherwise missed. Furthermore, we highlighted, in our photos, all clinicopathological signs of the optic nerve which suggested the presence of glaucoma (Figure 1). This result proves that this smartphone-based screening strategy could be improved by adding other tests, in future studies, such as tonometry and FDT visual field. On this basis, the transpalpebral applanation tonometer seems to be most suitable, in our context with limited resources, due to its low maintenance and acquisition costs, easy manipulation by inexperienced hands, its mobility, reproducibility and its hygienic nature (no contact with eye secretions). Li Y et al. recommend this in glaucoma screening strategies [15]. Performing FDT visual field, on the other hand, could be done in a cost-effective and reliable way with the use of a smartphone. The results obtained would be similar to those obtained by Humphrey, according to Lester et al [7].

The limitations of our study included low participation rate at the confirmatory examination with a lost-to-follow-up rate of 64% (25/39). This could be due to the fact that the confirmatory ophthalmological examination was not free of charge (expensive) and performed at a site different from the screening site. This is however not uncommon in community glaucoma screening strategies, where the rate of people lost-to-follow-up is sometimes over 40%, even when the confirmation examinations are free of charge [4, 5, 16]. Therefore, for future studies, reducing the cost of confirmatory examinations, using the same site for glaucoma screening and confirmation, using smartphones for telemedicine to be closer to the patient coupled with education may serve as effective public health strategies to improve glaucoma screening and diagnosis in resource depleted settings. 

## CONCLUSION

Using a smartphone with the MIIRetCam accessory makes it possible to archive all retinal photographs showing qualitative changes in the optic nerve, in glaucoma disease. Therefore, in a glaucoma screening approach, improving the quality of results and follow-up rate, would require the use of a contextualised methodology and integration of additional tests.
